# Effect of First‐Line Chemotherapy Alone Versus First‐Line Chemotherapy Plus Radiotherapy on Survival in Patients With Locally Advanced Pancreatic Cancer in the IMRT Era: A Retrospective Cohort Study

**DOI:** 10.1002/cnr2.70531

**Published:** 2026-04-01

**Authors:** Zihao Liu, Yaru Tian, Ji Ma, Dong‐Fang Meng

**Affiliations:** ^1^ Department of Interventional Radiology Shandong Cancer Hospital and Institute, Shandong First Medical University and Shandong Academy of Medical Sciences Jinan P. R. China; ^2^ Department of Radiation Oncology Shandong Cancer Hospital and Institute, Shandong First Medical University and Shandong Academy of Medical Sciences Jinan P. R. China; ^3^ Department of Oncology The First Affiliated Hospital of Shandong First Medical University and Shandong Provincial Qianfoshan Hospital, Shandong Key Laboratory of Rheumatic Disease and Translational Medicine, Shandong Lung Cancer Institute Jinan P. R. China

**Keywords:** chemoradiotherapy, chemotherapy, IMRT, locally advanced pancreatic cancer, survival outcomes

## Abstract

**Background:**

The role of intensity‐modulated radiotherapy (IMRT) and novel combination chemotherapy regimens in locally advanced pancreatic cancer (LAPC) remains unclear. In this study, we focused on comparing survival between first‐line chemotherapy alone and first‐line chemotherapy plus IMRT in patients with LAPC.

**Methods:**

A total of 70 patients from Shandong Cancer Hospital and Institute were enrolled. The primary endpoint was progression‐free survival (PFS). Survival outcomes were estimated by the Kaplan–Meier method and compared by the log‐rank test, and the multivariate Cox proportional hazards model was used to estimate hazard ratios (HRs), 95% CIs, and independent prognostic factors.

**Results:**

The median PFS was 10.0 months in the chemotherapy alone group and 14.0 months in the chemotherapy plus IMRT group (*p* = 0.465). Patients who received chemotherapy alone had a median OS of 12.0 versus 21.0 months for patients who received chemotherapy plus IMRT (*p* = 0.156). The chemotherapy alone group had a disease control rate (DCR) of 33.33% (6 of 18), while the chemotherapy plus IMRT group had a DCR of 61.54% (32 of 52) (*p* = 0.038). The multivariate Cox regression model was used to adjust for potential prognostic factors. Shorter PFS was observed in men (*p* = 0.042), and those with a high serum level of CA 19‐9 (*p* = 0.047). Similarly, shorter OS was significantly associated with men (*p* = 0.010) and a high serum level of CA 19‐9 (*p* = 0.020). Grade 3–4 fatigue or asthenia, decreased appetite, neutrophil count decreased, AST increased, and ALT increased were predominant in the chemotherapy alone group.

**Conclusion:**

The combination of first‐line chemotherapy and IMRT improves the DCR rate (*p* = 0.038) and was well tolerated. However, first‐line chemotherapy plus IMRT had no significant difference in PFS (*p* = 0.465) and OS (*p* = 0.156) compared with chemotherapy alone.

## 1 Introduction

Pancreatic cancer is one of the most lethal malignant neoplasms, with a mortality rate of 6.35/100 000 in China [1]. The incidence rates and mortality rates have increased slightly in China in recent decades [2]. Surgical resection is currently the only curative option to achieve long‐term survival in patients with pancreatic ductal adenocarcinoma (PDAC) [3]. However, only 15%–20% of patients can feasibly undergo resection [4]. Locally advanced pancreatic cancer (LAPC) accounts for 30% of newly diagnosed cases and is unlikely to require surgery because of the invasion of adjacent critical vascular structures [5]. Patients with LAPC are generally considered incurable, and the goal of the treatment is to prolong survival, control progression, and improve the patients' quality of life (QOL) [6]. The current guidelines for LAPC consist of enrollment in clinical trials, chemotherapy alone, concurrent chemoradiotherapy or sequential chemoradiotherapy [3]. Chemotherapy is the mainstay of LAPC treatment because the majority of these patients will never convert to surgical resection, and the risk of distant metastasis is very high [7]. Current standard first‐line chemotherapy regimens for patients with LAPC include FOLFIRINOX, modified FOLFIRINOX, gemcitabine plus albumin‐bound paclitaxel.

Radiation is frequently used for LAPC because of the potential of radiotherapy to improve local tumor control and, in some cases, increase the chance of margin‐negative (R0) resection in patients who receive surgery. The role of radiation in patients with LAPC remains controversial and is being investigated in ongoing studies [8, 9]. The LAP07 trial enrolled 449 patients with LAPC who underwent treatment with chemoradiotherapy or chemotherapy after receiving gemcitabine with or without erlotinib [10]. In the chemoradiotherapy group, patients received three‐dimensional conformal radiation therapy (3D‐CRT) and concurrent capecitabine, and the prescribed doses of radiation were 54 Gy to the planning target volume (PTV) in 30 fractions [11]. The results revealed no significant difference in overall survival (OS) between chemoradiotherapy and chemotherapy alone. Nevertheless, chemoradiotherapy decreased the rates of local progression (32% vs. 46%, *p* = 0.03). A systematic review of 11 clinical trials demonstrated that survival was better with chemoradiotherapy than with radiotherapy alone, but chemoradiotherapy followed by chemotherapy did not show any survival benefit over chemotherapy alone [12].

Advances in radiation techniques, including stereotactic body radiation therapy (SBRT) and intensity‐modulated radiation therapy (IMRT), have improved the effectiveness and minimized radiation exposure of normal tissues [11, 13, 14]. A previous study reported that LAPC patients treated with IMRT combined with gemcitabine concurrent chemoradiotherapy had improved OS and locoregional progression‐free survival (LRPFS) without increased acute and late gastrointestinal toxicities in comparison with those patients who received concurrent gemcitabine with three‐dimensional conformal radiation therapy (3DCRT) [11]. However, the role of advanced radiotherapy techniques and novel combination chemotherapy regimens in LAPC remains unclear. In this study, we focused on comparing survival between first‐line chemotherapy plus IMRT and first‐line chemotherapy alone in patients with LAPC.

## 2 Methods

### 2.1 Data Collection and Study Design

In this retrospective cohort study, we used the specialized Digital Medical Record System, which includes pancreatic cancer cases diagnosed between May 2018 and November 2021 in the Shandong Cancer Hospital and Institute, Jinan, China. Patients were eligible for enrollment if they were aged 18 years or older; had pathologically confirmed pancreatic ductal adenocarcinoma; had locally advanced pancreatic cancer (AJCC 8th staging system), which was not amenable to resection; had received at least 16 weeks of continuous first‐line chemotherapy or first‐line chemotherapy plus radiotherapy; had an Eastern Cooperative Oncology Group (ECOG) Performance Status of 0 or 1; and had no other tumor types or serious noncancerous illnesses. Exclusion criteria were prior treatment with pancreatectomy, radiotherapy, chemotherapy, as well as previous immunotherapy. A total of 70 patients were included in these eligibility criteria. Our study was reviewed and approved by the Clinical Research Ethics Committee of Shandong Cancer Hospital and Institute (SDTHEC202512005), and informed consent forms were signed by all patients. We have de‐identified all patient details. The reporting of this study conforms to Strengthening the Reporting of Observational Studies in Epidemiology (STROBE) guidelines.

### 2.2 Therapy and Follow‐Up

All patients were restaged according to the eighth edition of the American Joint Committee on Cancer staging system. The routine diagnostic and staging workup included a complete medical history, physical examination, MRI, CT, as well as positron emission tomography/computed tomography, if necessary. The decision to pursue chemotherapy alone versus chemotherapy plus IMRT for each patient was determined through multidisciplinary team (MDT) evaluation, based on factors including tumor location, tumor size, and specific symptoms.

Indications for chemoradiotherapy included: (1) Encasement of the celiac axis and/or superior mesenteric artery; (2) severe invasion or occlusion of the superior mesenteric vein and/or portal vein; (3) large or irregular primary tumor volume; (4) the presence or high risk of specific symptoms (e.g., neuralgia, cranial nerve involvement).

In the first‐line chemotherapy alone group, first‐line chemotherapy was administered for at least 4 months to patients with LAPC. First‐line chemotherapy consisted of FOLFIRINOX, modified FOLFIRINOX (mFOLFIRINOX), gemcitabine plus albumin‐bound paclitaxel (GA), or other gemcitabine‐based combination chemotherapy.

In the first‐line chemotherapy plus IMRT group, the treatment modalities included: (1) Induction chemotherapy with any of the first‐line regimens followed by IMRT alone or concurrent chemoradiotherapy; (2) IMRT alone or concurrent chemoradiotherapy followed by adjuvant therapy. Concurrent chemotherapy regimens include gemcitabine, capecitabine, and tegafur/uracil (S‐1).

All patients underwent IMRT prescribed to deliver 50–54 Gy in 1.8–2.0 Gy/fraction to the planning target volume (PTV). The gross tumor volume (GTV) included the gross tumor and enlarged lymph nodes. Clinical target volume (CTV) was defined as GTV with a 1‐cm margin, respecting anatomical boundaries, such as the stomach, duodenum, and transverse colon. The planning target volume (PTV) encompassed setup variability and internal motion. The treatment plan was optimized so that ≥ 95% of the PTV received ≥ 95% of the prescription dose, while the internal clinical target volume (CTV) and gross tumor volume (GTV) were encompassed by higher isodose lines, receiving at least 100% of the prescribed dose.

Induction chemotherapy (IC) and adjuvant chemotherapy (AC) consisted of FOLFIRINOX/mFOLFIRINOX, gemcitabine plus albumin‐bound paclitaxel (GA), or other gemcitabine‐based combination chemotherapy. A dosage of 1000 mg/m^2^ of gemcitabine on day 1 of each 28‐day cycle, 825 mg/m^2^ of capecitabine twice daily, or 40–60 mg/day tegafur/uracil (S‐1) twice daily on days 1–14 of each 21‐day cycle was administered during concurrent radiotherapy.

Patients received first‐line treatment until disease progression or unacceptable toxic effects. After first‐line treatment, subsequent therapy included 1 or more of the following: subsequent lines of therapy and observation/supportive care. Tumor response was evaluated by investigators, according to response evaluation criteria in solid tumors (RECIST) version 1.1, with laboratory assessments every 1 month and CT scans every 2 months. Laboratory assessments included complete blood counts, chemistry panels, and serum carbohydrate antigen 19‐9 (CA19‐9).

### 2.3 Outcomes

Disease control rate (DCR) was the percentage of patients with LAPC whose therapeutic intervention has led to a complete response, partial response, or stable disease. Objective response rate (ORR) was the percentage of patients with LAPC whose therapeutic intervention has led to a complete response or partial response. The primary endpoint was progression‐free survival (PFS), which was defined as the time from diagnosis until disease progression or death from any cause; the secondary endpoint was overall survival (OS), which was defined as the time from diagnosis until death from any cause.

### 2.4 Statistical Analysis

Clinical characteristics were compared between patients who received chemotherapy plus IMRT or chemotherapy alone as first‐line treatment. Continuous variables were compared using the *t*‐test; categorical variables were compared using the *χ* ^2^ test. Survival outcomes were estimated by the Kaplan–Meier method and compared by the log‐rank test. We used a multivariate Cox proportional hazards model to estimate HRs, 95% CIs and independent prognostic factors. All tests were two sided, and *p* < 0.05 was considered statistically significant. Statistical analyses were performed using SPSS, version 22 (SPSS Inc., Chicago) and R, version 4.1.1 (R Foundation).

## 3 Results

### 3.1 Patient Baseline Characteristics

Between May 2018 and November 2021, a total of 70 patients from Shandong Cancer Hospital and Institute were enrolled. Among them, 18 (25.71%) were treated with first‐line chemotherapy alone and 52 (74.29%) were treated with first‐line chemotherapy plus IMRT. The median age was 60.5 years (interquartile range (IQR) 40.0–79.0), 28 (40.0%) of 70 enrolled patients were female, and the percentage of patients with ECOG performance status 0–1 was well balanced. Demographics and baseline characteristics, including tumor location, size, and differentiation, are listed in Table 1.

**TABLE 1:** Baseline characteristics.

Characteristic	Treatment modality no. (%)		
	Chemotherapy alone (*n* = 18)	Chemortherapy plus IMRT (*n* = 52)	*p* ^a^
Age, median (IQR), *y*	57	61	0.355
	40–74	44–79	
Sex			
Female	4 (22.22)	24 (46.15)	0.132
Male	14 (77.78)	28 (53.85)	
ECOG performance status			
0	8 (44.44)	28 (53.85)	0.492
1	10 (55.56)	24 (46.15)	
Smoking status			
No	12 (66.67)	44 (84.62)	0.101
Yes	6 (33.33)	8 (15.38)	
Baseline CA 19‐9 level, median (range), U/mL	217.9	213.5	0.795
	2.0–4741.0	0.6–10000.0	
Tumor location in the pancreas			
Head or neck	12 (66.67)	32 (61.54)	0.698
Body or tail	6 (33.33)	20 (38.46)	
Tumor size, median (range), mm	36.0	39.0	0.210
	14.0–73.0	16.0–110.0	
Stage^b^			
Ib	1 (5.56)	3 (5.77)	0.239
IIa	0 (0)	0 (0)	
IIb	3 (16.67)	2 (3.85)	
III	14 (77.78)	47 (90.38)	
Pancreatic pain			
No	6 (33.33)	10 (19.23)	0.219
Yes	12 (66.67)	42 (80.77)	

Abbreviations: CA 19‐9, cancer antigen 19‐9; ECOG, Eastern Cooperative Oncology Group; IQR, interquartile range.

^a^ *p* values were calculated using the *χ* ^2^ test for categorical variables and the Mann–Whitney test for continuous variables.

^b^ According to the eighth edition of the AJCC staging system.

### 3.2 Response to Treatment

**FIGURE 1:**
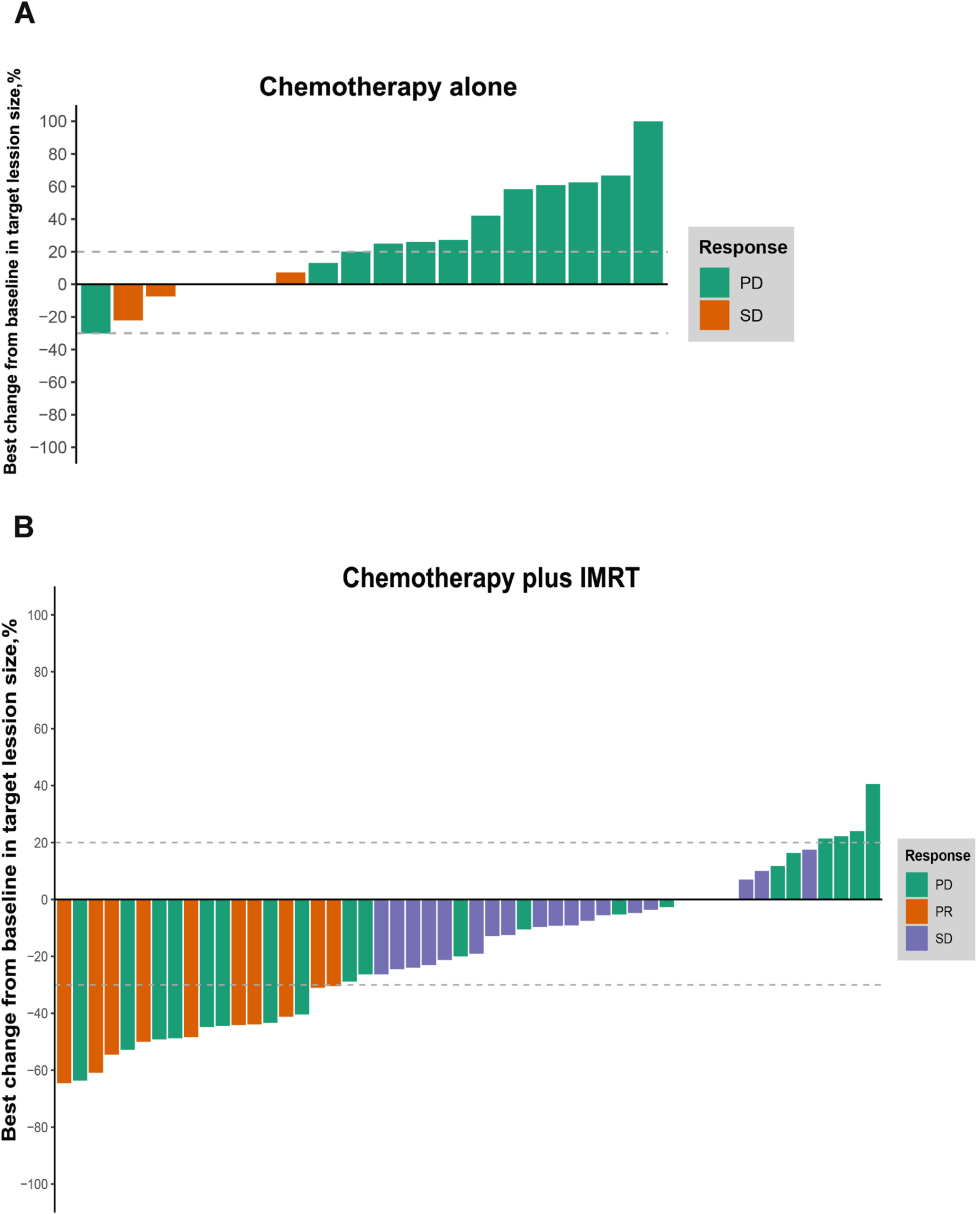
Tumor response in patients with locally advanced pancreatic cancer (LAPC). (A and B) Waterfall plots of tumor response in patients with LAPC. Dashed lines at 20% and −30% denote thresholds for progressive disease (PD) and partial response (PR), respectively, according to RECIST 1.1. PD, progressive disease; PR, partial response; SD, stable disease.

**FIGURE 2:**
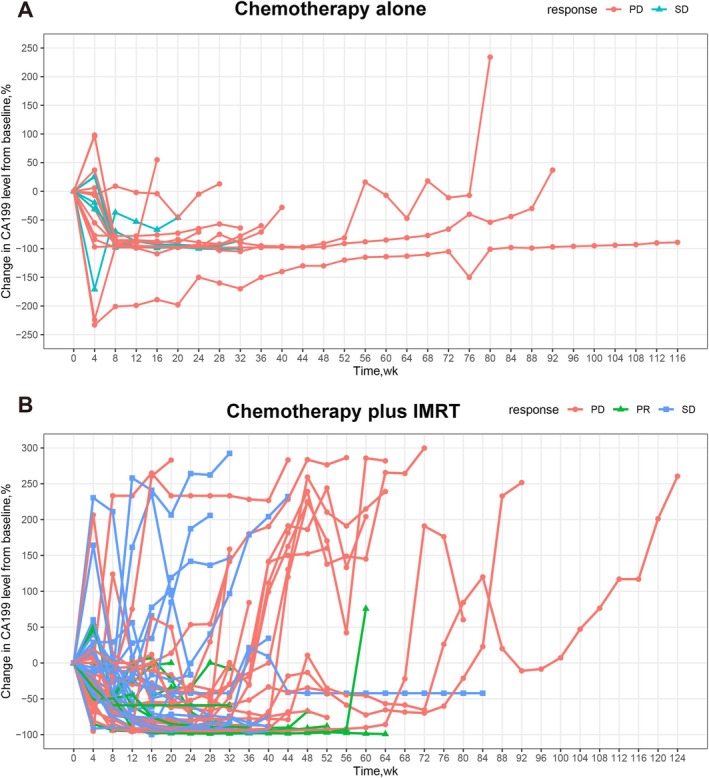
Cancer antigen 19‐9 (CA19‐9) response in patients with locally advanced pancreatic cancer (LAPC). (A and B) Spider plots show the percentage change of CA19‐9 from baseline over time. Wk, week.

All patients were evaluated for tumor response. For patients given chemotherapy alone, 6 (33.33%) had stable disease (SD), and 12 (66.67%) had progressive disease (PD), whereas in the chemotherapy plus IMRT group, 10 patients (19.23%) had a partial response (PR), 22 (42.31%) had SD, and 20 (38.46%) had PD (Figure 1). Overall, 0 (0%) of 18 patients in the chemotherapy alone group and 10 (19.23%) of 52 patients in the chemotherapy plus IMRT group achieved an overall objective response rate (ORR). The chemotherapy alone group had a disease control rate (DCR) of 33.33% (6 of 18), while the chemotherapy plus IMRT group had a DCR of 61.54% (32 of 52). We observed that the ORR was not significantly different between the chemotherapy alone group and the chemotherapy plus IMRT group (*p* = 0.054). However, compared to the chemotherapy plus IMRT group, patients treated with chemotherapy alone were less likely to achieve disease control (*p* = 0.038) (Figure 1). The dynamic levels of CA19‐9 during first‐line therapy using chemotherapy alone or chemotherapy plus IMRT are shown in a spider plot. All the patients with a baseline and at least one post‐baseline assessment, 12 (66.67%) patients in the chemotherapy alone group and 26 (50.00%) patients in the chemotherapy plus IMRT group had more than 50% declines of serum CA19‐9 concentrations in at least 20 weeks (Figure 2).

### 3.3 Survival Outcomes

**FIGURE 3:**
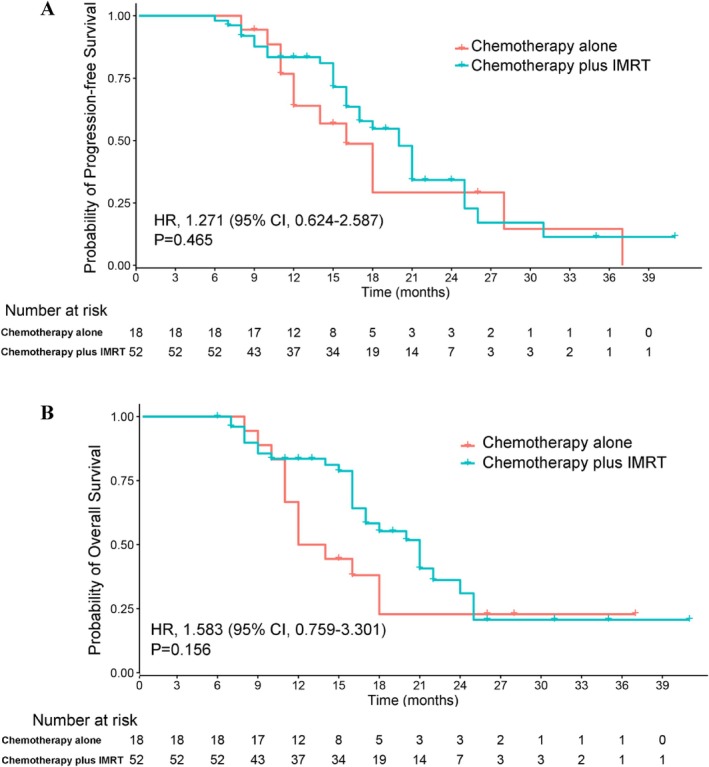
Kaplan–Meier plots for progression‐free survival (A) and overall survival (B). CI, confidence interval; HR, hazard ratio.

The median follow‐up time for the whole cohort was 15.0 months (range 2.0–41.0). At the end of the follow‐up period, 39 patients (55.71%) had died. The median PFS was 10.0 months (95% CI: 7.0–13.0 months) in the chemotherapy alone group and 14.0 months (95% CI: 11.5–16.5 months) in the chemotherapy plus IMRT group. The median follow‐up time for OS was 22.0 months (95% CI: 17.9–26.1 months). Patients who received chemotherapy alone had a median OS of 12.0 months (95% CI: 8.9–15.1 months) versus 21.0 months (95% CI: 17.5–24.5 months) for patients who received chemotherapy plus IMRT. There was no difference in PFS and OS between chemotherapy alone group and chemotherapy plus IMRT group (PFS, *p* = 0.465; OS, *p* = 0.156) (Figure 3).

### 3.4 Safety

Next we assessed the toxicity in the safety population (18 patients in the chemotherapy alone group; 52 patients in the chemotherapy plus IMRT group). The haematological and nonhaematological toxicities are shown in Table 2.

**TABLE 2:** Adverse events.

	Chemotherapy alone (*n* = 18)			Chemortherapy plus IMRT (*n* = 52)		
	Grade 1–2	Grade 3	Grade 4	Grade 1–2	Grade 3	Grade 4
Fatigue or asthenia	8 (44.4%)	2 (11.1%)	0	45 (86.5%)	0	1 (1.9%)
Nausea	7 (38.9%)	0	0	17 (32.7%)	0	0
Diarrhea	2 (11.1%)	0	0	5 (9.6%)	0	0
Decreased appetite	10 (55.6%)	2 (11.1%)	0	40 (76.9%)	2 (3.8%)	0
Constipation	2 (11.1%)	0	0	9 (17.3%)	0	0
Vomiting	6 (33.3%)	0	0	13 (25%)	0	0
WBC count decreased	10 (55.6%)	4 (22.2%)	0	32 (61.5%)	10 (19.2)	1 (1.9%)
Neutrophil count decreased	8 (44.4%)	4 (22.2%)	0	34 (65.4%)	5 (9.6%)	4 (7.7%)
Anemia	12 (66.7%)	1 (5.6%)	0	49 (94.2%)	2 (3.8%)	1 (1.9%)
Platelet count decreased	8 (44.4%)	1 (5.6%)	0	29 (55.8%)	4 (7.7%)	1 (1.9%)
AST increased	8 (44.4%)	2 (22.2%)	0	25 (48.1%)	0	0
ALT increased	8 (44.4%)	2 (22.2%)	0	25 (48.1%)	1 (1.9%)	0
Blood bilirubin increased	8 (44.4%)	1 (5.6%)	0	23 (44.2%)	3 (5.8%)	4 (7.7%)

*Note:* Data are *n* (%). All adverse events reported are shown. Some patients had more than one adverse event. Bold values represent a *p*‐value less than 0.05, indicating a statistically significant difference.

Abbreviations: ALT, alanine aminotransferase; AST, aspartate aminotransferase; WBC, white blood cell.

Any grade fatigue or asthenia (chemotherapy alone vs. chemotherapy plus IMRT: 55.5% vs. 88.4%), decreased appetite (66.7% vs. 80.7%), neutrophil count decreased (66.6% vs. 82.7%) and anemia (72.3% vs. 99.9%) were predominant in the chemotherapy plus IMRT group. By contrast, grade 3 or 4 fatigue or asthenia (11.1% vs. 1.9%), decreased appetite (11.1% vs. 3.8%), neutrophil count decreased (22.2% vs. 17.3%), AST increased (22.2% vs. 0%) and ALT increased (22.2% vs. 1.9%) were predominant in the chemotherapy alone group. No treatment‐related deaths occurred in both groups.

### 3.5 Multivariate Analysis

In the multivariate analysis, we used Cox regression analysis to adjust for potential prognostic factors, including age, sex, smoking, alcohol, ECOG PS, CA19‐9, stage, tumor site, tumor size, pancreatic pain, and treatment modality. The multivariable analysis is presented in Table 3. The results showed that shorter PFS was observed in men (HR 2.034 (1.027–4.029), *p* = 0.042), and those with a high serum level of CA 19‐9 (HR 2.328 (1.011–5.363), *p* = 0.047). Similarly, shorter OS was significantly associated with men (HR 2.664 (1.261–5.628), *p* = 0.010) and a high serum level of CA 19‐9 (HR 3.112 (1.195–8.101), *p* = 0.020) (Table 3).

**TABLE 3:** Multivariate analysis for prognostic factors in the 70 LAPC patients.

	Multivariable	
	HR (95% CI)	*p*
PFS		
Age		
< 60 years	Reference	0.303
≥ 60 years	0.715 (0.378–1.353)	
Sex		
Female	Reference	**0.042**
Male	2.034 (1.027–4.029)	
Smoking		
No	Reference	0.493
Yes	0.683 (0.230–2.030)	
Alcohol		
No	Reference	0.489
Yes	1.319 (0.602–2.890)	
ECOG PS		
0	Reference	0.725
1	1.147 (0.534–2.465)	
CA19‐9, U/mL		
< 35 normal	Reference	**0.047**
≥ 35 elevated	2.328 (1.011–5.363)	
Stage		
I–II	Reference	0.193
III	2.259 (0.663–7.695)	
Tumor site		
Body or tail	Reference	0.502
Head or neck	0.773 (0.365–1.637)	
Tumor size		
< 40 mm	Reference	0.472
≥ 40 mm	0.759 (0.358–1.608)	
Pancreatic pain		
No	Reference	0.187
Yes	0.604 (0.285–1.278)	
Treatment modality		
Chemotherapy alone	Reference	0.882
Chemotherapy plus IMRT	0.944 (0.442–2.018)	
OS		
Age		
< 60 years	Reference	0.911
≥ 60 years	0.960 (0.466–1.975)	
Sex		
Female	Reference	**0.010**
Male	2.664 (1.261–5.628)	
Smoking		
No	Reference	0.762
Yes	0.880 (0.385–2.010)	
Alcohol		
No	Reference	0.950
Yes	1.033 (0.373–2.858)	
ECOG PS		
0	Reference	0.882
1	1.080 (0.552–2.114)	
CA199, U/mL		
< 35 normal	Reference	**0.020**
≥ 35 elevated	3.112 (1.195–8.101)	
Stage		
I–II	Reference	0.988
III	0.992 (0.339–2.900)	
Tumor site		
Body or tail	Reference	0.435
Head or neck	1.332 (0.648–2.741)	
Tumor size		
< 40 mm	Reference	0.798
≥ 40 mm	0.909 (0.436–1.893)	
Pancreatic pain		
No	Reference	0.612
Yes	1.280 (0.493–3.325)	
Treatment modality		
Chemotherapy	Reference	0.156
Chemotherapy plus IMRT	0.607 (0.305–1.210)	

*Note: p* values were calculated using a Cox proportional hazard regression model with backward elimination.

Abbreviations: CA 19‐9, cancer antigen 19‐9; CI, confidence interval; DFS, disease‐free survival; ECOG PS, Eastern Cooperative Oncology Group performance status; OS, overall survival.

## 4 Discussion

Although chemoradiation is a conventional treatment modality for the management of LAPC, the utility of chemoradiation in LAPC patients is still debatable. In the LAP07 clinical trial, patients were initially randomized to receive gemcitabine alone or gemcitabine plus erlotinib. In the second randomization, patients who were not progressing received the same chemotherapy for 2 months or capecitabine‐based concurrent chemoradiotherapy (CCRT). There was no significant difference in PFS and OS with chemoradiotherapy compared with chemotherapy alone. However, chemoradiotherapy extended a longer period without treatment and decreased locoregional tumor progression [10]. The LAP07 study was designed before the advent of FOLFIRINOX/mFOLFIRINOX or GA regimen. Because the LAP07 study used outdated chemotherapy regimens, it lacks reliable evidence comparing which of the first‐line regimens is more effective. Accordingly, we evaluated first‐line chemotherapy alone versus first‐line chemotherapy plus IMRT in LAPC patients. The primary endpoints of PFS and OS showed no statistically significant difference between the chemotherapy alone group and the chemotherapy plus IMRT group in our study. Our findings which were supported by a pooled analysis of 11 trials demonstrated that chemotherapy plus IMRT did not increase OS compared with chemotherapy alone [10, 13, 15, 16, 17, 18, 19].

A major goal of chemoradiation in the unresectable setting is to enhance local control and delay disease progression. Although first‐line chemotherapy plus IMRT had no significant difference in PFS and OS compared with chemotherapy alone in our study, patients who received chemotherapy plus IMRT had a higher DCR rate than those who received chemotherapy alone. Chemoradiation can also be administered as palliative treatment for cancer pain that is refractory to analgesic therapy [20, 21]. A previously published meta‐analysis compared survival for patients who underwent CRT, RT alone or chemotherapy alone in the locally advanced setting [22]. CRT significantly improved long‐term survival compared to RT alone or chemotherapy alone. CRT also increased treatment‐related toxicities. In our study, the chemotherapy plus IMRT group had a higher incidence of TRAEs (treatment‐related adverse events) than the chemotherapy alone group, but they were all manageable. This increase was associated with higher incidences of fatigue or asthenia (55.5% vs. 88.4%), decreased appetite (66.7% vs. 80.7%), neutrophil count decreased (66.6% vs. 82.7%) and anemia (72.3% vs. 99.9%) in the chemotherapy plus IMRT group. The increased hematological toxicities were likely because of the combined use of chemotherapy and radiotherapy. Furthermore, most of the nonhaematological TRAEs that occurred in the chemotherapy alone group were grade ≥ 3. In particular, drug‐induced liver injury was significantly higher in the chemotherapy‐alone group compared to the chemoradiotherapy group, which may be attributed to the greater intensity of chemotherapy alone. Our results showed that chemotherapy combined with IMRT was well tolerated.

Gemcitabine has been used as a radiation sensitizer in LAPC [23]. Several studies suggest that gemcitabine‐based chemoradiation is associated with similar and even better survival than concurrent 5‐fluorouracil chemotherapy and radiotherapy [24]. The use of oral fluoropyrimidine drugs, including capecitabine and tegafur, has also been evaluated in LAPC and confirmed to be effective. The phase II SCALOP trial demonstrated an improvement in health‐related quality‐of‐life for patients with LAPC who received capecitabine‐based chemoradiation compared with gemcitabine‐based chemoradiation [25]. Feasibility of gemcitabine and fluoropyrimidine was generally recommended as a concurrent chemotherapy regimen in LAPC, with survival benefits compared with radiotherapy alone. Based on this, in our study, gemcitabine‐ and fluoropyrimidine‐based regimens were adopted as concurrent chemotherapy protocols to achieve radiotherapy potentiation.

The role of upfront chemoradiation in the management of LAPC has not been definitively established. The results of 3 phase II trials evaluated the upfront chemoradiation regimen in LAPC, with a median overall survival of 8.6 months [26]. Results from the phase 2 JCOG1106 trial demonstrated that S‐1‐based concurrent chemoradiotherapy had longer survival compared with gemcitabine‐based induction chemotherapy followed by chemoradiotherapy for LAPC [27]. The results of an ECOG trial comparing upfront chemoradiotherapy to chemotherapy in LAPC have been discussed [28]. Of 74 patients enrolled in this study who were assigned to receive gemcitabine alone (arm A) or gemcitabine‐based chemoradiation followed by gemcitabine alone (arm B), superior median overall survival was observed in arm B compared with arm A (11.1 vs. 9.2 months; HR, *p* = 0.017). Unfortunately, the study was terminated early because of the poor accrual rate; the PFS was not different between the 2 arms, and the confidence intervals (CIs) for median OS were widened and overlapped. These results did not appear to provide sufficient evidence to determine the standard of therapy [29]. The phase III FFCD‐SFRO study also assessed the upfront chemoradiation compared with chemotherapy alone in patients with LAPC. The analysis of data showed that 1‐year OS was significantly longer in the gemcitabine alone arm of the study (53% vs. 32%; HR, 0.54; 95% CI: 0.31–0.96; *p* = 0.006). The study was ended prematurely, because intention to treat (ITT) analysis revealed that patients in the chemoradiation arm had a lower survival rate and severe toxicity, indicating that the differences in survival outcomes were probably due to toxicity related to the chemoradiation treatment [30]. Despite the ambiguous results of the ECOG and FFCD‐SFRO trial, upfront chemoradiation therapy is still an option for selected patients with uncontrolled pain or local invasion with bleeding.

Currently, chemoradiation or SBRT following chemotherapy is used in the LAPC setting. Patients receive 2–6 cycles of induction chemotherapy before proceeding to chemoradiation if patients have good performance status and no developed metastatic disease. The goal of induction chemotherapy in patients with LAPC is to improve systemic disease control. In addition, induction chemotherapy increases the likelihood of selecting those patients who are more responsive to therapy so as to benefit from subsequent chemoradiation. Retrospective data from the GERCOR studies demonstrated that after control of disease by first‐line chemotherapy, subsequent chemoradiation therapy had a significant survival benefit compared with chemotherapy alone [31]. However, results from the SCALOP and LAP‐07 clinical trials in patients with unresectable PDAC suggested that chemoradiation following chemotherapy had no survival benefit compared to chemotherapy alone [32].

Multiple technological advancements in radiotherapy, including IMRT, magnetic resonance‐guided adaptive radiotherapy (MRgART), proton therapy and carbon ion therapy, allow radiation oncologists to reduce normal tissue doses while precisely targeting tumor regions [33, 34, 35]. IMRT is widely applied for the treatment of LAPC. The use of IMRT increases the RT dose to the gross tumor and reduces the toxicity to surrounding normal tissues [36]. A retrospective study aimed to compare the efficacy of IMRT and three‐dimensional conformal radiotherapy (3DCRT) in patients with LAPC. The results showed that IMRT improved OS and locoregional progression‐free survival (LRPFS) compared with 3DCRT. In addition, acute and late gastrointestinal toxicities of grade 2 or greater were more frequent with 3D‐CRT than with IMRT (*p* = 0.042; *p* = 0.47, respectively) [37]. A systematic review including 13 original studies of IMRT and 7 3DCRT studies found no significant difference in OS and PFS between IMRT and 3DCRT in patients with pancreatic cancer [37]. However, nausea, vomiting, diarrhea and gastrointestinal hemorrhage occurred more commonly in the 3DCRT group than in the IMRT group, suggesting that IMRT is well tolerated and improves the ability to deliver higher radiation doses to tumor regions [37].

Although patients with LAPC rarely undergo resection for curative purposes, advances in surgical techniques and the increasing use of neoadjuvant combination regimens have extended the pool of patients who may benefit from surgical resection [38]. The most critical aspects of neoadjuvant therapy are downstaging to a resectable or borderline resectable status, increasing the possibility of margin‐negative R0 resection [39]. After neoadjuvant therapy, surgical resection rates range from 12% to 60% [40, 41]. Recently, the CONKO‐007 trial assessed chemotherapy alone compared with induction chemotherapy (IC) followed by gemcitabine‐based chemoradiotherapy in patients with nonresectable LAPC [42]. The first results demonstrated that IC plus CRT improved the R0 circumferential resection margin (CRM)‐negative resection and pathologic complete response (pCR) rates. However, the primary endpoints of R0 resection rates, PFS, and OS were not significantly different in the whole cohort [43]. Of note, in our study, patients who responded well to initial therapy did not undergo surgical resection due to the rare tumor downstaging rate and surgical conversion rate.

The definition of resectability for LAPC depends on the level of surgeons and the willingness to perform complex surgical procedures. Given the prognosis associated with pancreatectomy and postoperative complications, screening patients is necessary, and those cases should be carefully discussed in high‐volume centers with multidisciplinary expertise [23]. In the absence of evident recommendations, surgical resection is considered an optimal therapy for LAPC patients with decreased CA 19‐9 levels, good ECOG performance status, and no disease progression after 4–6 months of neoadjuvant therapy [3].

## 5 Limitations

We acknowledge that our study has several limitations. First, a major limitation of our study is the significant sample size imbalance between the treatment groups: specifically, 18 patients received first‐line chemotherapy alone, compared with 52 patients who received first‐line chemotherapy plus IMRT. This disparity, inherent to the retrospective and single‐center design with a limited overall enrollment, may affect the robustness and generalizability of the comparative findings. Second, because of the retrospective study design, we did not distinguish the sequencing of chemoradiation and eliminate the heterogeneity of chemotherapy regimen and duration. Third, due to the limited sample size, performing rigorous subgroup analyses based on specific chemotherapy regimens (e.g., AG vs. FOLFIRINOX) or sequencing would lack statistical power. Further multicenter trials are needed to comprehensively evaluate our observations.

## 6 Conclusion

In conclusion, we found that the combination of first‐line chemotherapy and IMRT improves the DCR rate. However, first‐line chemotherapy plus IMRT had no significant difference in PFS and OS compared with chemotherapy alone.

## Author Contributions

**Zihao Liu:** methodology, writing – original draft, software, investigation. **Yaru Tian:** conceptualization. **Ji Ma:** data curation, supervision. **Dong‐Fang Meng:** writing – review and editing, formal analysis, funding acquisition, visualization.

## Funding

This work was supported by Shandong Provincial Natural Science Foundation of China (ZR2021QH208 to Dongfang Meng, ZR2021QH245 to Yaru Tian); National Natural Science Foundation of China (82303508 to Dongfang Meng, 82103632 to Yaru Tian).

## Ethics Statement

This study was reviewed and approved by the Clinical Research Ethics Committee of Shandong Cancer Hospital and Institute, and informed consent was signed by all patients.

## Conflicts of Interest

The authors declare no conflicts of interest.

## Data Availability

The datasets are not available for public access due to patient privacy concerns but are available from the corresponding author on reasonable request.
